# Rehabilitation and therapeutic exercise in obstructive sleep apnea: a perspective beyond the apnea–hypopnea index

**DOI:** 10.3389/fmed.2026.1873380

**Published:** 2026-09-16

**Authors:** Luis F. Guerrero-Vega, Víctor R. Jáuregui-Gutiérrez, Jonathan Elías-León, David A. Trejo-Osorio, Orlando R. Pérez-Nieto, Jonathan Reyes-Montufar, María del Carmen García-Pantoja, Fernando Mendiola-Enriquez, Cesar E. Vargas-Reynoso, Rafael A. Reyes-Monge, Silvana Macarena-Liendro, Mariana I. Razo-Alvarado, María de la Luz Paniagua-Sosa, Emanuel Olvera-Montoya, Abraham M. Tolentino-de la Mora, Edwin E. Herrera-Estrada, Luis A. Morgado-Villaseñor, Alonso J. Muñoz-Velázquez, Dante A. Linton-Cornejo

**Affiliations:** 1 Escuela Superior de Medicina, Instituto Politécnico Nacional, Mexico City, Mexico; 2 Department of Physical Therapy and Rehabilitation, Hospital General San Juan del Río, Querétaro, Mexico; 3 Intensive Care Unit, Hospital General San Juan del Río, Querétaro, Mexico; 4 Universidad Anáhuac Querétaro, Queretaro, Mexico; 5 Universidad Autónoma de Querétaro, Querétaro, Mexico; 6 Department of Pulmonary Medicine, Hospital General San Juan del Río, Querétaro, Mexico; 7 Universidad del Valle de Puebla, Puebla, Mexico; 8 UNE Universidad de Especialidades, Guadalajara, Mexico; 9 Intensive Care Unit, Hospital de la Mujer, Michoacan, Mexico; 10 Department of Pulmonary Medicine, Hospital Privado de Cordoba, Córdoba, Argentina; 11 Universidad Nacional de Cordoba, Córdoba, Argentina; 12 Universidad del Valle de Mexico, Queretaro, Mexico; 13 Instituto Nacional de Enfermedades Respiratorias, Mexico City, Mexico; 14 Unidad Mexico Americana del Golfo, Puebla, Mexico; 15 Intensive Cardiovascular Care Unit, Instituto Nacional de Cardiologia Ignacio Chavez, Mexico City, Mexico; 16 Universidad Marista de Valladolid, Michoacan, Mexico; 17 Department of Physical Therapy and Rehabilitation, Hospital general de León, Guanajuato, Mexico; 18 Benemérita Universidad Autonóma de Puebla, Puebla, Mexico

**Keywords:** obstructive sleep apnea, rehabilitation, exercise therapy, physiotherapy, apnea-hypopnea index, sleep quality, functional capacity, continuous positive airway pressure

## Abstract

**Introduction:**

Obstructive sleep apnea (OSA) is a multisystem disease whose clinical impact extends beyond nocturnal respiratory events. Although the apnea hypopnea index (AHI) is widely used to classify disease severity, this parameter does not adequately reflect the functional, symptomatic, and quality-of-life burden experienced by individuals with OSA. In this context, rehabilitative interventions have emerged as relevant therapeutic strategies within the comprehensive management of the disease.

**Objective:**

To synthesize the available scientific evidence on the role of rehabilitation in individuals with OSA, highlighting its clinical impact beyond the AHI and its contribution to patient-centered outcomes.

**Methods:**

A narrative review with structured bibliographic search was conducted in PubMed, Embase/Scopus, and Google Scholar. Randomized controlled trials, systematic reviews, and meta-analyses evaluating rehabilitative interventions in adult populations with OSA were included. The search covered exercise training, oropharyngeal myofunctional therapy, inspiratory muscle training, and interdisciplinary lifestyle programs using a comprehensive, multi-term search syntax.

**Results:**

The evidence indicates that rehabilitative interventions lead to consistent improvements in daytime sleepiness, sleep quality, functional capacity, cognitive performance, and quality of life, even when changes in the AHI are modest or variable. Interdisciplinary programs demonstrated the most robust effects, including reductions in AHI exceeding 50% in selected subgroups (INTERAPNEA trial). A recent umbrella review confirmed that structured physical activity achieved the greatest improvements in quality of life among all non-pharmacological interventions evaluated.

**Conclusion:**

Rehabilitation represents an essential component of the comprehensive management of OSA, providing clinically meaningful benefits beyond the AHI and reinforcing a therapeutic approach centered on patient functionality and quality of life.

## Introduction

Obstructive sleep apnea (OSA) is a sleep-related breathing disorder characterized by recurrent episodes of partial or complete upper airway obstruction, leading to intermittent hypoxia, sleep fragmentation, and sustained sympathetic activation. Its global prevalence has increased significantly over recent decades, in parallel with rising obesity rates; however, OSA also affects non-obese individuals, in whom anatomical, neuromuscular, and ventilatory control mechanisms play a relevant role.

Traditionally, the evaluation and treatment of OSA have focused on the apnea–hypopnea index (AHI) as the primary marker of disease severity and therapeutic response. Nevertheless, accumulating evidence indicates that this polysomnographic parameter does not adequately reflect the functional and symptomatic burden of the disease. Multiple studies have demonstrated a poor correlation between changes in AHI and clinically relevant outcomes, such as daytime sleepiness, quality of life, cognitive performance, and social participation, even following interventions that normalize nocturnal respiratory events.

From a broader clinical perspective, OSA should therefore be understood as a multisystem disease, with metabolic, cardiovascular, neurocognitive, emotional, and functional consequences. Individuals living with OSA frequently experience excessive daytime fatigue, impaired attention and memory, reduced physical performance, mood disturbances, and a marked decline in quality of life. In addition, non-restorative sleep is associated with musculoskeletal pain, reduced social participation, and decreased work productivity.

Within this framework, rehabilitation and physiotherapy have emerged as fundamental components of comprehensive OSA management. Interventions such as exercise training, oropharyngeal myofunctional therapy, inspiratory muscle training, and interdisciplinary lifestyle modification programs have demonstrated consistent benefits in functional and symptomatic outcomes that are meaningful to patients, even when their effects on the AHI are modest or variable. A recent umbrella review of meta-analyses confirmed that structured physical activity achieved the greatest improvements in quality of life among all non-pharmacological OSA interventions evaluated, underscoring its central role in patient-centered care.

Despite the growing body of evidence, methodological heterogeneity and substantial variability in intervention protocols and outcome measures persist, limiting comparability across studies and hindering clinical translation. Accordingly, the objective of this narrative review is to analyze the scientific evidence on rehabilitative interventions in individuals with OSA, highlighting their impact beyond the AHI and emphasizing functional and patient-centered outcomes that more accurately reflect the clinical burden of the disease.

## Study design

This work is classified as a narrative review with a structured bibliographic search, consistent with published guidance on narrative review methodology and with the scope of the research question. The present review does not fulfill the criteria of a formal systematic review under PRISMA 2020 standards, as it was not prospectively registered and did not perform formal risk-of-bias assessment using validated instruments across all domains. This constitutes a limitation acknowledged in the Limitations section of the Discussion.

Given the substantial clinical and methodological heterogeneity of the available evidence on rehabilitative interventions in OSA, a narrative approach was deliberately chosen to enable an integrative synthesis across multiple intervention modalities (exercise, oropharyngeal myofunctional therapy, inspiratory muscle training, and interdisciplinary programs), which would be difficult to synthesize within a single homogeneous quantitative framework.

### Data sources

The bibliographic search was performed in three electronic databases: PubMed/MEDLINE, Embase (via Scopus), and Google Scholar. These sources provide complementary coverage of biomedical and clinical literature relevant to OSA rehabilitation, including indexed peer-reviewed journals, grey literature, and conference proceedings.

### Search period

The search covered literature published from January 2000 to April 2026, with no language restriction for title/abstract screening. Only articles available in full text in English were included for qualitative synthesis.

### Search strategy

A structured multi-term search was performed using Boolean combinations of controlled vocabulary terms and free-text keywords. The expanded search syntax used was:

*(“obstructive sleep apnea” OR “OSA” OR “sleep disordered breathing”) AND (“exercise” OR “aerobic training” OR “resistance training” OR “physical activity” OR “physiotherapy” OR “rehabilitation” OR “myofunctional therapy” OR “oropharyngeal exercises” OR “inspiratory muscle training” OR “respiratory muscle training” OR “lifestyle intervention” OR “weight loss program”).*

This expanded syntax was developed to correct a limitation identified during peer review: the original three-term syntax (“exercise AND OSA AND physiotherapy”) did not adequately capture the literature on oropharyngeal myofunctional therapy and respiratory muscle training, which are central concepts analyzed in this review. The revised syntax ensures complete coverage of all intervention modalities discussed.

The search yielded a total of 103 records across the three databases after deduplication. Titles and abstracts were screened independently, followed by full-text review to determine eligibility.

Inclusion Criteria

Randomized controlled trials, systematic reviews, and meta-analyses.

Studies conducted in adult populations (≥ 18 years) diagnosed with OSA.

Research evaluating physiotherapeutic interventions or exercise-based programs as part of OSA management.

Articles published in the English language.

Studies reporting at least one of the following outcomes: AHI, ESS, PSQI, VO_2_max, quality of life, or functional capacity.

Article Selection and PRISMA Flow

Of the 103 records identified, 54 were excluded after title and abstract screening (irrelevant population, intervention, or outcomes). Of the remaining 49 records, 33 were excluded after full-text review for the following reasons: absence of a control group (*n* = 8); outcomes limited to AHI without functional measures (*n* = 7); non-adult population (*n* = 4); protocol papers without results (*n* = 5); duplicate or superseded data (*n* = 5); insufficient methodological detail to enable synthesis (*n* = 4). Eighteen studies were ultimately included for qualitative analysis. The study selection process is depicted in Figure 1 (PRISMA flow diagram).

**FIGURE 1:**
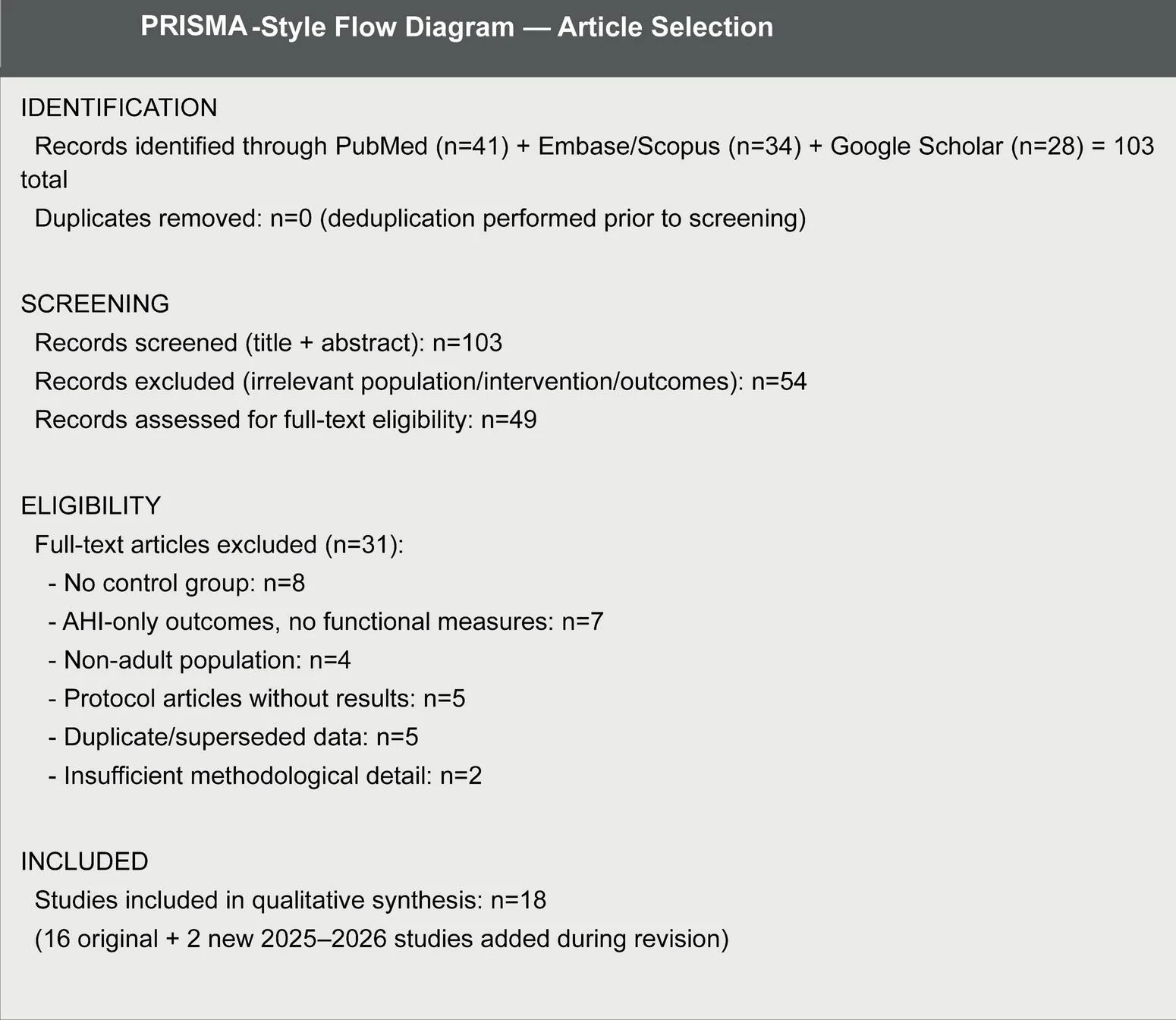
PRISMA-Style flow diagram — article selection.

Note: The original manuscript reported 16 included studies. Two additional studies published in 2025–2026 identified during the revised search (1, 2) were incorporated, bringing the total to 18 (Supplementary Table 1).

## Physiopathology

Obstructive sleep apnea (OSA) is a highly prevalent sleep disorder characterized by recurrent episodes of upper airway collapse during sleep, resulting in intermittent hypoxia, hypercapnia and sleep fragmentation. The core pathophysiological defect is the inability of upper airway dilator muscles, particularly the genioglossus, to adequately counteract the negative intraluminal pressure generated during inspiration (3–5). This failure stems from an unfavourable balance between collapsing forces (increased by anatomical narrowing) and protective dilator muscle activity, which decreases markedly at sleep onset and is further reduced during REM sleep. The Starling resistor model elegantly explains upper airway behaviour: when downstream pressure falls below a critical closing pressure (Pcrit), the pharynx becomes prone to flow limitation and collapse (6).

Anatomical factors play a central role in raising collapsibility. Obesity leads to fat deposition around the tongue and pharyngeal walls, craniofacial anomalies (retrognathia, micrognathia) and soft-tissue abnormalities (enlarged tonsils, low-lying palate) narrow the oropharyngeal lumen, and nocturnal rostral fluid shift in the supine position (especially in heart failure and renal disease) increases extravascular tissue pressure around the airway (7–9). These structural features are often clinically evident by a high Mallampati score or cephalometric abnormalities and are exacerbated by the supine posture and nasal obstruction.

Neuromuscular control is equally critical. In healthy individuals, upper airway dilator muscles activate phasically just before diaphragmatic contraction, stiffening and enlarging the airway. In OSA patients, baseline dilator muscle tone is higher during wakefulness (compensating for the narrow airway) but falls excessively during sleep, particularly in REM, leading to inadequate compensation for the increased negative pressure (10). Ventilatory control instability (high loop gain), low arousal threshold and impaired genioglossus responsiveness further perpetuate cyclic obstruction–arousal cycles, creating a self-sustaining pattern of respiratory instability (11).

Recurrent intermittent hypoxia and sleep fragmentation trigger widespread systemic consequences: oxidative stress, sympathetic overactivity, systemic inflammation, endothelial dysfunction, gut microbiota dysbiosis and metabolic dysregulation (12). These mechanisms explain the strong bidirectional associations between OSA and cardiometabolic diseases (hypertension, insulin resistance, atrial fibrillation, heart failure), neurocognitive impairment and increased risk of stroke, cancer and even severe COVID-19 (13).

Contemporary understanding has evolved toward an integrative, multifactorial model in which OSA is viewed as a spectrum of phenotypic traits or “endotypes” (anatomical collapsibility, muscle responsiveness, loop gain and arousal threshold) (14). This framework moves away from a one-size-fits-all approach and supports precision medicine strategies that target the dominant pathophysiological driver(s) in each patient, paving the way for personalised therapies beyond conventional continuous positive airway pressure.

## Limitations of the AHI-centered biomedical model

Multiple studies have demonstrated that obstructive sleep apnea (OSA) cannot be adequately understood or evaluated solely on the basis of the apnea–hypopnea index (AHI). Although this polysomnographic parameter quantifies the frequency of respiratory events during sleep, it fails to comprehensively capture the functional, symptomatic, and psychosocial burden experienced by individuals living with OSA.

In a detailed analysis, Weaver et al. observed that classic respiratory indices, including the AHI, do not consistently correlate with changes in daytime sleepiness, quality of life, or neurocognitive performance, either before or after surgical interventions or placebo treatments. These findings underscore the limited ability of the AHI to represent the true clinical impact of OSA (15). Similarly, Kirkham et al. reported a substantial discordance between changes in AHI and patient-perceived changes in symptoms and quality of life, even in contexts where treatment objectively modified nocturnal respiratory patterns (16).

Furthermore, studies such as that by Lee et al. have shown that quality of life in individuals with OSA is much more closely associated with daytime sleepiness, non-restorative sleep, and depressive symptoms than with AHI severity (17). This dissociation between polysomnographic severity and the patient’s clinical experience has driven the development of disease-specific instruments such as the Functional Outcomes of Sleep Questionnaire (FOSQ) and the Sleep Apnea Quality of Life Index (SAQLI), which were specifically designed to assess functional and participation domains that the AHI does not capture (18, 19).

Taken together, this body of evidence supports a central premise: OSA is a multisystem disease whose impact extends beyond nocturnal respiratory events. Consequently, an evaluation focused exclusively on the AHI is reductionist and limits the identification of clinically meaningful outcomes. This reinforces the need for evaluative and therapeutic approaches that prioritize patient-relevant outcomes, integrating functional, symptomatic, and quality-of-life dimensions into the assessment and management of OSA (Figure 2).

**FIGURE 2:**
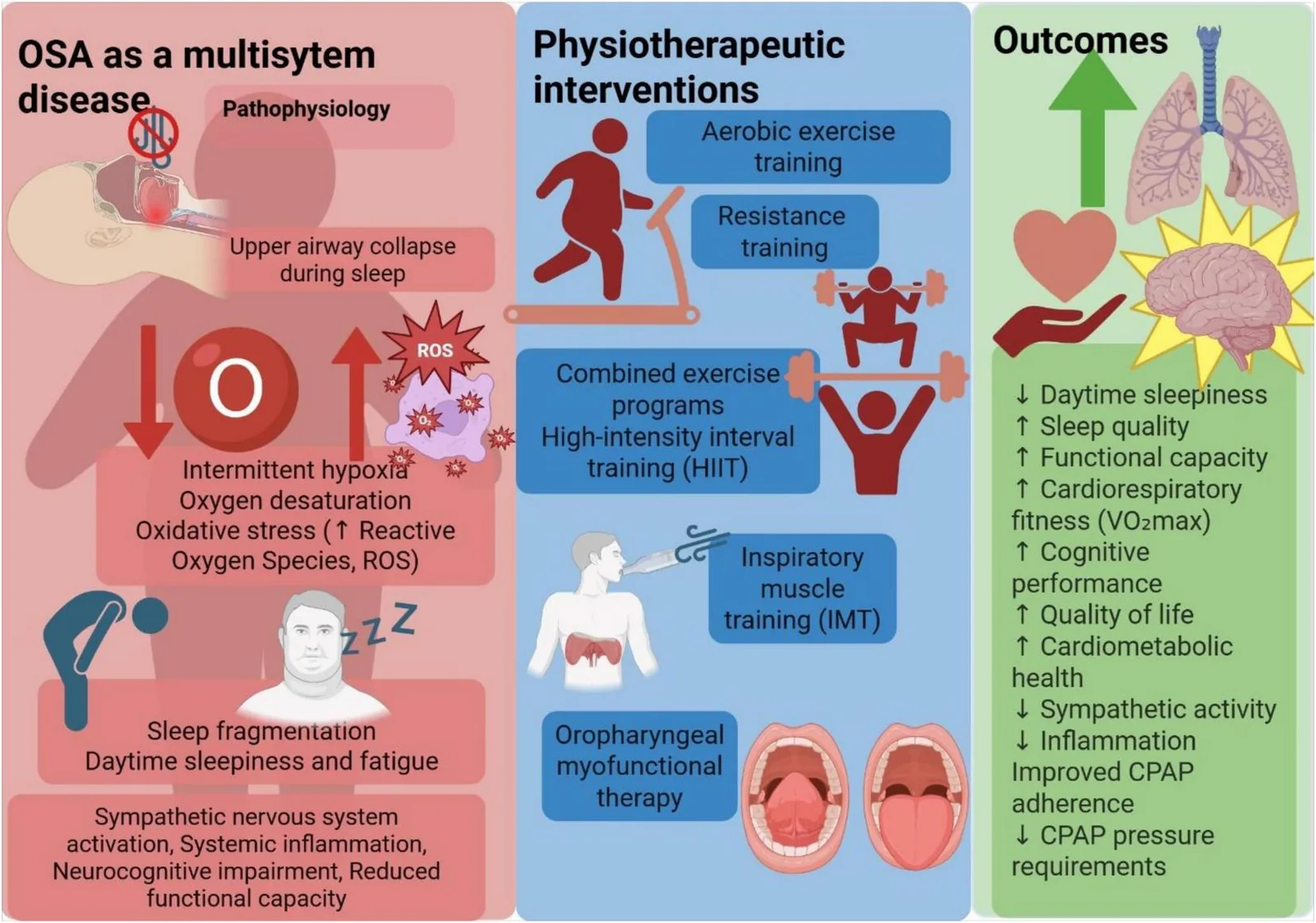
Rehabilitation-based approach in obstructive sleep apnea beyond the apnea–hypopnea index. Obstructive sleep apnea (OSA) is depicted as a multisystem disease characterized by upper airway collapse, intermittent hypoxia, oxidative stress, systemic inflammation, sleep fragmentation, and functional impairment. Physiotherapeutic and rehabilitation-based interventions—including aerobic and resistance exercise, inspiratory muscle training, oropharyngeal myofunctional therapy, and lifestyle modification—target key pathophysiological mechanisms beyond nocturnal respiratory events. These interventions are associated with clinically meaningful improvements in daytime sleepiness, functional capacity, cardiorespiratory fitness, quality of life, and cardiometabolic health, while producing modest and variable changes in the apnea–hypopnea index (AHI). Rehabilitation strategies act as complementary therapies to continuous positive airway pressure (CPAP), supporting a patient-centered and function-oriented model of care for OSA. Created with BioRender.com.

## Exercise-based rehabilitation

Accumulating evidence indicates that exercise training constitutes an effective rehabilitative intervention in OSA, with clinical benefits that extend beyond modifications in nocturnal respiratory parameters and encompass functionally relevant domains such as daytime sleepiness, cardiorespiratory capacity, and sleep quality.

A systematic review and meta-analysis by Peng et al. confirmed that aerobic or combined exercise programs produce a modest but statistically significant reduction in AHI, while also yielding consistent improvements in daytime sleepiness (ESS), sleep quality (PSQI), and cardiorespiratory fitness (VO_2_max) (20). A network meta-analysis by Tang et al. identified combined exercise training as one of the most effective non-instrumental strategies for reducing AHI and improving daytime sleepiness (21).

A subsequent umbrella review of meta-analyses of OSA interventions confirmed that, while CPAP remains the most effective intervention for AHI reduction, exercise-based rehabilitation achieves the largest improvements in health-related quality of life across all modalities evaluated (22). These findings align with evidence from individual RCTs, including Kline et al. and Sengul et al., which demonstrated significant reductions in daytime sleepiness and improvements in functional capacity following supervised aerobic exercise programs (23, 24).

From a dose-response perspective, Iftikhar et al. demonstrated that maximum AHI effects are observed when weekly aerobic exercise reaches approximately 100 min per week, with larger effects observed in studies with 12 or more weeks of training (25). This provides actionable guidance for clinical protocol design (Table 1).

**TABLE 1:** Summary of clinical studies evaluating rehabilitation-based interventions in individuals with obstructive sleep apnea.

Author (reference)	Title / Design	Location	*N*	Intervention	Key results
Pawar (40)	Systematic Review and Meta-analysis	Europe	*N* = 594	Aerobic programs (graduated walking, HIIT 2–3 × /week)	AHI: MD -5.24 (*p* < 0.00001). VO_2_max: MD + 5.84 (*p* = 0.03)
Sevugaperumal et al. (33)	Comparative study	India	*N* = 20	Group A: Myofunctional + IMT. Group B: IMT only. 12 weeks, 50 min/day, 7 days/week	Group A: PSQI pre 16.3→post 5.1; AHI pre 16.3→post 8.0 (*p* < 0.05). Group B: PSQI pre 15.9→post 10.2; AHI pre 16.9→post 13.7 (*p* < 0.05)
Atef et al. (41)	Prospective study	Egypt	*N* = 18	Moderate-intensity interval training (MIIT), 3 × /week for 10 weeks	Improvements in sleep latency, total sleep time, sleep efficiency, PSQI (*p* < 0.001), 6MWD (*p* = 0.008)
Martínez Revuelta et al. (42)	Systematic Review and Meta-analysis	Spain	*N* = 16 studies	Aerobic vs. combined aerobic + resistance programs	Combined aerobic + resistance: significant AHI reduction (*p* < 0.01)
Iftikhar et al. (25)	Systematic Review and Meta-analysis	USA	*N* = 435	Supervised aerobic training (moderate–high intensity)	Max AHI effect at ∼100 min/week: MD -10.92. ≥ 12 weeks: MD -15.94
Lins-Filho et al. (43)	RCT	Brazil	*N* = 39	12-week HIIT vs. control	AHI: HIIT -8.6 vs. control + 8.2 (*p* = 0.005). VO_2_max: + 4.8% (*p* = 0.001)
Carneiro-Barrera et al. (32)	RCT (INTERAPNEA)	Spain	*N* = 89	Interdisciplinary: exercise + weight loss + sleep hygiene + CPAP, 8 weeks	AHI -51% at 8 weeks, -57% at 6 months. 62% CPAP-free; 29% OSA remission
da Silva et al. (44)	RCT	Brazil	*N* = 23	12-week resistance training, 2 × 1 h/week, progressive cycles	REI decreased 3.6 events/h (95% CI -0.7 to -5.4)
Peng et al. (20)	Systematic Review and Meta-analysis	China	*N* = 444	Aerobic (4 studies), aerobic + resistance (5 studies)	AHI: MD -6.65 (*p* < 0.00001). Combined: MD -7.36. Aerobic only: MD -4.36
Dar et al. (45)	Systematic Review and Meta-analysis	India	*N* = 7 studies	IMT with resistive devices, 30–75% MIP, 5–45 min/session	Improved MIP, sleep quality, and daytime sleepiness across included studies
Karlsen et al. (46)	RCT (24-week follow-up)	Norway	*N* = 45	HIIT + strength training, 3 × /week (weeks 0–12), then advice (weeks 12–24)	AHI: between-group difference -10.4 events/h at 24 weeks (*p* = 0.016). VO2peak: + 4.0 mL/kg/min (*p* < 0.001)
Ueno-Pardi et al. (47)	RCT	Brazil	*N* = 47	Supervised exercise training, 3 × 60 min/week, 6 months	Decreased AHI, improved attention/executive function, increased cerebral glucose metabolism (*p* < 0.05)
Goya et al. (48)	RCT	Brazil	*N* = 48	72 sessions over ∼40 weeks. Aerobic + strength, 60 min	VO2peak + 4.7 (*p* < 0.05). AHI -7.4 (*p* < 0.05). Improved executive performance and MSNA reduction
Jurado-Garcia et al. (49)	RCT	Spain	*N* = 70	6-month progressive home walking program vs. usual care	AHI -∼6 points; greater benefit in severe OSA. HDL improved; LDL decreased
Hall et al. (50)	Cross-sectional	Canada	*N* = 155,448	Physical activity (MET-hours/week) assessed by self-report	Each unit increase in MET-hours/week: OR 0.98 (95% CI 0.96–1.00) for OSA (*p* ≤ 0.045)
Mônico-Neto M et al. (51)	Longitudinal cohort	Brazil	*N* = 658	Physical activity assessed by IPAQ (active vs. inactive)	Active: RR = 0.877 for OSA development. Active with OSA: halved risk of T2DM (RR = 0.493)
**Díaz de Terán et al.** (2) **★NEW**	RCT	Spain/ International *N* = 32	OMT + cervical spine exercises, 20 weeks, single-blind	Significant improvements in AHI, ODI, TC90, daytime sleepiness (ESS), and quality of life (EQ-5D) vs. control (*p* < 0.05)	
**Torres-Castro et al.** (1) **★NEW**	Overview of Systematic Reviews	International	*N* = 23 SRs	IMT, various devices and protocols, multiple respiratory conditions	OSA: IMT increased MIP by 29.6 cmH_2_O (95% CI 6.0–53.1). Confirmed consistent effect on inspiratory muscle strength.

The table describes study design, geographic location, sample characteristics, type of intervention, and main clinical outcomes, including respiratory indices, functional performance, sleep quality, and patient-reported outcomes. Interventions include aerobic and combined exercise training, inspiratory muscle training, oropharyngeal myofunctional therapy, and interdisciplinary lifestyle programs. Outcomes are reported using both polysomnographic measures and functional or symptom-based endpoints to reflect the multidimensional impact of rehabilitation in obstructive sleep apnea. AHI, Apnea–Hypopnea Index; BMI, Body Mass Index; CMRgl, Cerebral Metabolic Rate of Glucose; CG, control group; ES, effect size; ESS, Epworth Sleepiness Scale; FEV1, Forced Expiratory Volume in One Second; FITT, Frequency, Intensity, Time, and Type; FOSQ, Functional Outcomes of Sleep Questionnaire; HDL, High-Density Lipoprotein; HIIT, High-Intensity Interval Training; HOMA-IR, Homeostatic Model Assessment of Insulin Resistance; IPAQ, International Physical Activity Questionnaire; IG, Intervention Group; PImax, Maximal Inspiratory Pressure; HRmax, Maximum Heart Rate; MD, Mean Difference; MET-hours/week, Metabolic Equivalent Task–hours per week; MSNA, Muscle Sympathetic Nerve Activity; OSA, obstructive sleep apnea; OR, Odds Ratio; VO2peak, Peak Oxygen Uptake; SpO_2_, Peripheral Oxygen Saturation; PSQI, Pittsburgh Sleep Quality Index; RCT, Randomized Controlled Trial; REI, Respiratory Event Index; SAQLI, Sleep Apnea Quality of Life Index; SD, Standard Deviation; SMD, Standardized Mean Difference; SCWT, Stroop Color and Word Test; HRtraining, Training Heart Rate; VLDL, Very Low-Density Lipoprotein; VO_2_max, Maximal Oxygen Uptake; 95% CI, 95% Confidence Interval. Bold values indicate the primary outcome of interest in each study: either AHI reduction or the main functional/symptom-based endpoint reported as the primary result by the original authors.

## Oropharyngeal myofunctional therapy

Oropharyngeal myofunctional therapy represents another rehabilitative intervention with growing evidence in the management of obstructive sleep apnea (OSA). The randomized controlled trial by Guimarães et al., considered a seminal study in this field, demonstrated that 3 months of targeted exercises focusing on the tongue, soft palate, and pharyngeal muscles were associated with an approximately 38% reduction in the apnea–hypopnea index (AHI), along with significant improvements in daytime sleepiness, sleep quality, and snoring intensity, compared with a placebo group (23).

Concordant findings were reported in the randomized controlled trial by Ieto et al., in which the application of oropharyngeal exercises was associated with reduced snoring and clinical symptom improvement in patients with mild to moderate OSA (26). In an integrative manner, a systematic review conducted by de Felício et al. included these and other studies, concluding that myofunctional therapy consistently reduces AHI, daytime sleepiness, and nocturnal awakenings, while also improving health-related quality of life. However, the authors highlighted that the available evidence is still based on relatively small samples and heterogeneous protocols, which limits the generalizability of the findings (27).

From a clinical perspective, myofunctional therapy appears to be particularly useful in mild to moderate OSA, as well as a complementary strategy to continuous positive airway pressure (CPAP) in patients with adherence difficulties. Overall, this intervention provides an anatomical–functional rehabilitative approach supported by clinical evidence, with particular relevance due to its ability to improve symptoms and functional outcomes without reliance on device-based therapies, aligning with patient-centered models of care. Oropharyngeal myofunctional therapy represents a rehabilitative intervention with growing evidence in OSA management. The randomized controlled trial by Guimarães et al. demonstrated that 3 months of targeted exercises focusing on the tongue, soft palate, and pharyngeal muscles were associated with an approximately 38% reduction in AHI, along with significant improvements in daytime sleepiness, sleep quality, and snoring intensity (28). Ieto et al. reported concordant findings regarding snoring reduction (26).

A recent RCT by Díaz de Terán et al. (2) evaluated the combination of oropharyngeal myofunctional therapy and cervical spine exercises over 20 weeks in adults with mild-to-moderate OSA, demonstrating significant improvements in AHI, oxygen desaturation index, daytime sleepiness, and quality of life compared to control extending the evidence base for this intervention to a combined physiotherapeutic approach (2).

A systematic review by de Felício et al. concluded that myofunctional therapy consistently reduces AHI, daytime sleepiness, and nocturnal awakenings, while improving health-related quality of life (27). Myofunctional therapy appears to be particularly useful in mild-to-moderate OSA, as well as a complementary strategy to CPAP in patients with adherence difficulties, providing an anatomical–functional rehabilitative approach that aligns with patient-centered models of care.

## Respiratory muscle training

Inspiratory muscle training (IMT) has emerged as a promising rehabilitative intervention for individuals living with obstructive sleep apnea (OSA). In a randomized controlled trial, Azeredo et al. demonstrated that 12 weeks of IMT with progressive loads (40–70% of maximal inspiratory pressure [MIP]) were associated with significant increases in inspiratory muscle strength, along with clinically relevant improvements in daytime sleepiness and sleep quality, as well as a reduction in the apnea–hypopnea index (AHI), compared with a control group that trained with minimal load (29).

Complementarily, feasibility studies and smaller clinical trials have shown that IMT is a safe, well-tolerated, and feasible intervention, with consistent benefits in inspiratory muscle strength and subjective sleep perception in individuals with OSA (30). These findings suggest that strengthening the inspiratory musculature may favorably influence relevant pathophysiological mechanisms, such as upper airway stability and ventilatory control during sleep.

From a comparative perspective, the network meta-analysis conducted by Tang et al. indicated that, although respiratory muscle training exerts a moderate effect on AHI, it produces significant improvements in daytime sleepiness and subjective sleep quality, positioning it as a valid and complementary rehabilitative alternative to other non-instrumental interventions (21).

Taken together, this evidence suggests that IMT may contribute to improved ventilatory stability and respiratory control during sleep, particularly when integrated into multimodal rehabilitation programs focused on functional outcomes and patient-centered clinical experiences.

## Interdisciplinary lifestyle and rehabilitation programs

Comprehensive rehabilitation programs that combine exercise training, body weight management, health education, and optimization of sleep habits have demonstrated some of the most robust results in the therapeutic management of obstructive sleep apnea (OSA). An early review by Shneerson already highlighted that weight reduction, together with improvements in sleep habits—such as establishing regular sleep schedules, avoiding alcohol and sedatives, optimizing the sleep environment, and incorporating physical exercise—could reduce OSA severity, although complete normalization was rarely achieved (31).

The most compelling example of this approach is the INTERAPNEA trial, conducted by Carneiro-Barrera et al., in which an intensive eight-week program focused on structured exercise, weight loss, sleep hygiene, and reduction of alcohol and tobacco consumption was added to continuous positive airway pressure (CPAP) treatment in 89 men with moderate to severe OSA. The results were clinically remarkable: the program achieved a 51% reduction in AHI at 8 weeks and a 57% reduction at 6 months, whereas the control group exhibited minimal changes. Furthermore, 62% of participants no longer required CPAP at 6 months, and 29% achieved complete remission of OSA, accompanied by significant improvements in quality of life and cardiometabolic parameters (32).

Concordant results have been reported in interventions centered on the Mediterranean diet and broader lifestyle modification programs, in which reductions in AHI and symptoms were sustained even without changes in CPAP therapy (33, 34). Consistently, accumulated evidence suggests that weight loss of ≥ 10–15% can induce profound improvements and even OSA remission in a subset of patients (35, 36).

Taken together, these findings position interdisciplinary rehabilitation as an intervention capable of modifying core pathophysiological factors of OSA, beyond symptom control or exclusive reliance on device-based therapies. This reinforces the need for integrated, patient-centered therapeutic models focused on clinically meaningful outcomes.

## Relationship between rehabilitation and CPAP

Although CPAP remains the most effective intervention for normalizing AHI and nocturnal oxygen saturation, rehabilitative therapies provide fundamental complementary benefits that extend beyond the control of nocturnal respiratory parameters (37). A network meta-analysis by Iftikhar et al. demonstrated that non-instrumental interventions such as exercise training achieve improvements in daytime sleepiness of a magnitude clinically comparable to those obtained with mandibular advancement devices (38).

The review of OSA treatment in the modern era (39) synthesizes evidence supporting a paradigm shift: non-CPAP interventions, including rehabilitative strategies, have demonstrated substantial effects on patient-centered outcomes that CPAP alone does not fully address, particularly quality of life and daytime functioning (39). This narrative further reinforces the need to move beyond AHI normalization as the sole treatment goal.

The umbrella review by Papageorgiou et al. (37) confirmed that, across 230 RCTs and multiple meta-analyses, CPAP and exercise should be viewed as complementary strategies: CPAP is superior in AHI and ESS reduction, while exercise achieves the greatest quality-of-life improvements (22). Specific components of rehabilitation, including oropharyngeal myofunctional therapy and lifestyle modification programs, have also shown potential to improve CPAP adherence, reduce required therapeutic pressures, or facilitate CPAP withdrawal in selected subgroups.

## Discussion

The reviewed evidence highlights that, although the AHI is an essential parameter for classifying the respiratory severity of OSA, it does not adequately reflect the functional and symptomatic burden experienced by individuals living with this condition. Clinical studies by Weaver et al. and Kirkham et al. have documented that changes in AHI correlate poorly or inconsistently with clinically relevant outcomes, including daytime sleepiness, quality of life, and cognitive performance, even following therapeutically effective interventions from a polysomnographic standpoint (15, 16).

Within this context, rehabilitative interventions demonstrate consistent benefits precisely in these functional domains. The umbrella review, which synthesized evidence from 230 RCTs across multiple meta-analyses, confirmed that structured physical activity achieved the greatest quality-of-life improvements among all non-pharmacological OSA interventions a finding that directly positions exercise as a first-line therapeutic strategy for patient-centered outcomes (22). Recent meta-analyses by Peng et al. and Tang et al. similarly confirm that aerobic or combined exercise is associated with significant improvements in daytime sleepiness, sleep quality, and functional capacity, even when AHI reductions are modest (20, 21, 52).

The RCT by Díaz de Terán et al. (2) advances the evidence for oropharyngeal myofunctional therapy by demonstrating that a combined OMT and cervical spine exercise program sustained over 20 weeks produced significant benefits in AHI, oxygenation, daytime sleepiness, and quality of life in mild-to-moderate OSA—extending the scope of myofunctional rehabilitation beyond isolated tongue and palate exercises (2). This finding is complemented by the overview of IMT reviews by Ribas-Serna et al. (22), which confirmed a consistent 29.6 cmH_2_O increase in MIP with IMT in OSA, providing strong evidence for respiratory muscle strengthening as a physiologically meaningful intervention (1).

Finally, interdisciplinary rehabilitation programs provide the strongest evidence that OSA is a highly modifiable condition when multiple pathophysiological determinants are addressed simultaneously. The INTERAPNEA trial demonstrated AHI reductions exceeding 50%, along with sustained quality-of-life improvements, through an intervention integrating exercise training, weight loss, and health education (32). The network meta-analysis by Zhang et al. (53) corroborates these findings at the level of combined modalities, while the narrative synthesis by Farre et al. (39) frames this evolution as a paradigm shift in OSA management—from AHI normalization to comprehensive, patient-centered care (39, 53).

## Limitations

This work has several limitations that must be acknowledged. First, as a narrative review, it does not fulfill all criteria of a formal systematic review and is subject to potential selection bias in the included literature. A prospective registration in PROSPERO was not performed. Second, despite the expanded search strategy, only three databases were used; additional sources such as Cochrane CENTRAL, CINAHL, or Web of Science may contain relevant studies not captured here. Third, risk-of-bias assessment using validated instruments (Cochrane RoB 2.0 or GRADE) was not conducted across all included studies, limiting the strength of causal inference. Fourth, the heterogeneity in intervention modalities, outcome measures, and follow-up periods across included studies precludes formal meta-analytic synthesis within this review. Future systematic reviews should prioritize standardized protocols and comprehensive risk-of-bias appraisal.

## Conclusion

The available evidence indicates that rehabilitation provides significant clinical and functional value in the management of OSA, beyond the modifications observed in the AHI. Rehabilitative interventions—including exercise training, oropharyngeal myofunctional therapy, inspiratory muscle training, and interdisciplinary lifestyle programs—are consistently associated with reductions in daytime sleepiness, improvements in physical and cognitive function, and a meaningful increase in health-related quality of life.

A recent umbrella review of over 230 RCTs on OSA interventions confirms that structured physical activity achieves the greatest quality-of-life improvements among all non-pharmacological modalities evaluated, definitively repositioning rehabilitation as a first-line component of patient-centered OSA management—not merely an adjunct to CPAP therapy.

Emerging evidence from 2025 to 2026, including new RCTs on combined oropharyngeal and musculoskeletal approaches and updated overviews of IMT, further strengthens the mechanistic rationale for multimodal rehabilitation: neuromuscular control of the upper airway, ventilatory stability, cardiorespiratory conditioning, and patient-perceived functional outcomes can all be favorably modified through rehabilitation, independently of significant AHI reduction.

These findings support the need to position physiotherapy and rehabilitation as central components of interdisciplinary OSA management, integrated in a complementary manner with standard therapies, and oriented toward functionality, quality of life, and outcomes that truly matter to patients.

## Recommendations for future research

Standardize rehabilitative intervention protocols for exercise training, oropharyngeal myofunctional therapy, and respiratory muscle training by more precisely defining optimal modality, intensity, frequency, and duration.

Conduct formal systematic reviews with prospective registration, multi-database searches, and GRADE-level evidence appraisal to provide the highest-quality synthesis of rehabilitation evidence in OSA.

Design pragmatic clinical trials in real-world clinical settings to evaluate rehabilitative interventions under routine clinical practice conditions.

Incorporate comprehensive outcome assessments combining polysomnographic measures, daytime symptoms, physical performance, cognitive function, and PROMs.

Conduct long-term studies to analyze the durability of rehabilitative interventions, including adherence, persistence of functional benefits, and interaction with standard therapies such as CPAP.

Identify patient subgroups that may derive differential benefit from rehabilitation based on OSA severity, cardiometabolic profile, functional phenotype, or CPAP adherence, advancing toward a personalized medicine approach.

Evaluate multimodal and interdisciplinary strategies comparing isolated versus combined interventions to determine therapeutic synergies and establish optimal integration models.

## Supplementary material

Supplementary Table 1 Complete search strings used in PubMed/MEDLINE, Embase (via Scopus), and Google Scholar, including Boolean operators, MeSH terms, free-text keywords, language filters, and date ranges (January 2000 - April 2026). Provided for methodological transparency and replicability.
