# Supplementary material for: Rehabilitation and therapeutic exercise in obstructive sleep apnea: a perspective beyond the apnea–hypopnea index
Source: Front Med (Lausanne). 2026 Sep 16;13:1873380. doi: 10.3389/fmed.2026.1873380 (PMC13624400; doi:10.3389/fmed.2026.1873380)
Supplement: Supplementary file 1 [file Data_Sheet_1.pdf]

Author and title	Type	Location	Population	Intervention	Results
Pawar M, (2025) Effectiveness of aerobic exercise training in patients with obstructive sleep apnea: a systematic review and meta-analysis[36].	Systematic Review and Meta-analysis	Europe	N=594	Jurado-García et al., 2020: Graduated walking program, 5 times/week for 24 weeks. Intensity monitored using the Borg Rating of Perceived Exertion scale (levels 9–15). Progressive increase in walking time from 20 to 45 minutes per session. Karlsen et al., 2017: High-intensity interval training (HIIT) twice/week for 12 weeks. Work intervals at 90–95% of maximum heart rate, with warm-up and cool-down at 70% of maximum heart rate. Lins-Filho et al., 2023: HIIT three times/week for 12 weeks. Four-minute cycles walking or running at 90–95% of maximum heart rate interspersed with 3 minutes walking at 50–55% of maximum heart rate.	Aerobic training produced a significant reduction, mean difference (MD) −5.24 ($p<0.00001$), indicating fewer respiratory events during sleep. VO_2max: Significantly improved, MD 5.84 ($p=0.03$), reflecting a substantial improvement in aerobic capacity.

Sangeetha Sevugaperuma, et al. (2025) Effectiveness of myofunctional therapy along with inspiratory muscle training versus inspiratory muscle training on severity and quality of sleep among an OSA subjects[37].	Comparative study	India	N=20	Group A: Myofunctional therapy plus IMT. Myofunctional therapy included exercises to strengthen the tongue, soft palate, and pharyngeal muscles to improve airway stability and function. IMT aimed to improve inspiratory muscle strength and endurance to optimize respiratory function and reduce airway collapse. Group B: IMT only. Both groups performed 50-minute sessions daily, 7 days/week, for 12 weeks	Group A: PSQI significantly improved (pre 16.30±2.67 vs post 5.10±1.29; t=11.6208; $p<0.05$). AHI significantly reduced (pre 16.30±5.10 vs post 8.00±3.30; t=7.7175; $p<0.05$).<br>Group B: PSQI significantly improved (pre 15.90±2.33 vs post 10.20±2.20; t=21.8943; $p<0.05$). AHI significantly reduced (pre 16.90±4.63 vs post 13.70±4.45; t=8.9134; $p<0.05$).
Atef H, et al. (2024) Comparative effects of moderate-intensity interval training on sleep quality and functional capacity in atrial fibrillation patients with two types of sleep apnea[38].	Prospective Study	Egipt	N=18	Three exercise sessions/week for 10 weeks (30 sessions total), with good adherence. Intensity prescribed according to the FITT principle (American College of Sports Medicine). Work phases: moderate intensity, target	Significant differences were observed in: sleep latency ($p<0.001$), total sleep duration ($p=0.026$), sleep efficiency ($p<0.001$), PSQI ($p=0.001$), and 6-minute walk distance (6MWD) ($p=0.008$).

				heart rate 55–75% of HRmax. Recovery phases: low intensity, target heart rate 45–50% of HRmax. Interval training consisted of 4 moderate-intensity bouts interspersed with 3 low-intensity bouts.	
Martínez Revuelta L, et al. (2024) Relationship Between Obstructive Sleep Apnea and Sports Systematic Review and Meta-Analysis[39].	Systematic Review and Meta-analysis	Spain	N= 16 studies	Aerobic exercise only: programs involving aerobic activity alone (including aerobic exercise and home-based exercise). Combined aerobic and resistance training: programs integrating aerobic exercise with resistance training.	Participants in programs combining aerobic and resistance exercise experienced a significant reduction in AHI; $p<0.01$, indicating high statistical significance.
Iftikhar IH, et al (2024), Aerobic exercise training and obstructive sleep apnea: dose-response meta-analyses[40].	Systematic Review and Meta-analysis	USA	N= 435	Supervised aerobic exercise training, moderate to high intensity. Session duration ranged from 30 to 90 minutes. Intensity: 90–95% HRmax for higher-intensity intervals and 50–55%	Maximum AHI effects were observed when weekly aerobic exercise reached ~100 min/week: MD −10.92 (95% CI −15.57 to −6.27). When restricted to studies with ≥12 weeks of aerobic

				HRmax for lower-intensity intervals.	training, larger effects were observed: MD −15.94 (95% CI −23.72 to −8.15) with only 70 min/week.
Lins-Filho, O. L., et al. (2023) "Effect of high-intensity interval training on obstructive sleep apnea severity: A randomized controlled trial"[41].	RCT	Recife, Brazil	N=39	12-week allocation to: HIIT intervention group vs control group (no active intervention detailed; likely usual care).	AHI: control increased by 8.2 points, whereas HIIT improved by −8.6 points (p=0.005). Oxygenation: control decreased −1.6% vs HIIT +0.4% (p=0.030). Total sleep time: control −31 min vs HIIT +31 min (p=0.049). Sleep efficiency: control −3.2% vs HIIT +9.9% (p=0.026). VO_2max: control −1.1 vs HIIT +4.8% (p=0.001).
Carneiro-Barrera, A., Amaro-Gahete, F. J., et al. (2022). "Effect of a Weight Loss and Lifestyle Intervention on Dietary Behavior in Men with Obstructive Sleep Apnea: The INTERAPNEATrial"[29].	RCT	Spain	N=89 men	Nutritional education (Mediterranean diet), exercise program, sleep hygiene, smoking/alcohol cessation + usual CPAP care. Eight weeks, 3 supervised sessions/week, 60–70 minutes each..	The intervention group showed significantly greater improvement in dietary behavior vs control: between-group mean difference +8.7 points in FBC score (95% CI)

Silva RP, et al. (2022) Effect of short-term resistance training on the respiratory event index in older patients with obstructive sleep apnea. [42]	RCT	Brazil	N=23	12-week resistance training, two 1-hour sessions/week (24 sessions total). Progressive program in three 4-week cycles with increasing intensity/volume (12–14 reps → 10–12 reps → 8–10 reps to transient concentric failure). Abdominal exercises progressed from 2×10 to 3×20 reps.	REI decreased by 3.6 events/hour (95% CI −0.7 to −5.4) in the exercise group; effect size 0.3. Body mass changes were small; significant group×time interaction reported for strength outcomes; sit-to-stand time decreased by −2.7 seconds
Li S, Sun Z, Liu Y, et al.(2022) Effect of Exercise Intervention on Obstructive Sleep Apnea: A Systematic Review and Meta-Analysis[18].	Systematic Review and Meta-analysis	China	N: 444	Four studies used aerobic exercise protocols; five combined aerobic plus resistance exercise. Modalities: treadmill or stationary cycling, brisk outdoor walking, traditional Chinese exercises (e.g., tai chi). Intensity predominantly low to moderate for aerobic exercise. Resistance training used light weights with	AHI: overall reduction MD −6.65 (95% CI −7.77 to −5.53), $p<0.00001$; overall finding ≈5.95 reduction in AHI. Subgroups: combined aerobic+resistance MD −7.36 (95% CI −8.64 to −6.08), $p<0.00001$; aerobic only MD −4.36 (95% CI −6.67 to −2.06), $p=0.0002$. Minimum SaO_2: MD +1.67 (95% CI 0.82 to 2.52), $p=0.0001$.

				multiple repetitions. Frequency ranged from 3 to 6 sessions/week	VO_2peak: SMD 0.54 (95% CI 0.31 to 0.78), p<0.00001.
Dar JA (2022) Effect of inspiratory muscle training on inspiratory muscle strength, sleep quality, daytime sleepiness, lung function, and exercise capacity in obstructive sleep apnea: a systematic review and meta-analysis. [43]	Systematic Review and Meta-analysis	India	N= 7 studies	Main intervention: IMT using resistive devices. Load ranged from 30% to 75% of maximal inspiratory pressure (PImax). Session duration ranged from 5 to 45 minutes. La duracion Varió entre 5 y 45 minutos máximo por sesión.	Main intervention: IMT using resistive devices. Load ranged from 30% to 75% of maximal inspiratory pressure (PImax). Session duration ranged from 5 to 45 minutes.
Karlsen, T., et al. (2022). [44]	RCT (follow-up)	Norway	N=51 baseline; 24-week follow-up N=45	Intervention group: supervised exercise weeks 0–12 (3 sessions/week), combining HIIT + strength training vs usual care; weeks 12–24 both groups received exercise advice for unsupervised continuation to evaluate maintenance.	AHI: significant improvement in the intervention group at 12 weeks and maintained at 24 weeks; between-group difference at week 24: −10.4 events/hour in favor of exercise, p=0.016. VO_2peak: improved at 12 weeks and maintained at 24 weeks; +4.0 mL/kg/min in favor of exercise, p<0.001.

Ueno-Pardi LM, , et al. (2022) Effects of exercise training on brain metabolism and cognitive functioning in sleep apnea [45]	RCT	Brazil	N=47	Supervised exercise training (ET), 3 sessions/week, 60 minutes each for 6 months. Each session: 5-min warm-up; cycling 25 min in month 1, increasing to 40 min in months 2–6; 10-min strength exercises; 5-min cool-down with stretching.	T group showed improved exercise capacity; decreased AHI, oxygen desaturation index, and arousal index ($p<0.05$); significant increase in CMRgl vs control ($p<0.05$); improved attention and executive function, with positive correlation between increased CMRgl and improved Stroop test performance ($p<0.05$). Inverse associations between post-ET CMRgl and AHI severity ($r=-0.43$), microarousal index ($r=-0.53$), and oxygen saturation changes with attention test time ($r\approx-0.43$ to -0.46), $p<0.05$.
Goya TT, et al. (2021), Exercise training reduces sympathetic nerve activity and improves executive	RCT	Brazil	N=48	72 sessions completed over ~40±3.9 weeks. Frequency: 1–3 days/week. Session (60 min): 5-min stretching; 40-	VO_2peak increased in exercise group (+4.7±0.8) vs control (−1.2±0.4), $p<0.05$. AHI decreased in exercise

performance in individuals with obstructive sleep apnea. [46]				min cycle ergometer at heart-rate intensity between anaerobic threshold and respiratory compensation point (from maximal cardiopulmonary exercise test); 10-min strengthening (RPE 5–7/10) for trunk and lower/upper body muscles; 5-min cool-down.	group (−7.4±3.1) vs control (+5.5±3.3), $p<0.05$. MSNA decreased at rest and during SCWT after training; executive performance improved (12.4% increase in correct responses during SCWT); no change in control group
Jurado-Garcia et al., (2020). "E ectofaGraduated Walking Program on the Severity of Obstructive Sleep Apnea Syndrome. ARandomized Clinical Trial" [47]	RCT	Spain	N=70	Intervention group (n=29): usual care plus a 6-month home-based progressive walking program	AHI decreased by ~6 points vs control; greater benefit in severe vs moderate OSA (45 vs 34 events/hour; p=0.002). Oxygen desaturation index improved (43.3 vs 34.3/h; p=0.046). HDL increased (45.3 vs 49.5 mg/dL; p=0.009). LDL decreased (141.2 vs 127.5 mg/dL; p=0.038).
Hall KA, et al. (2020). Physical activity is associated with reduced prevalence of self-reported obstructive sleep	Cross-sectional study	Ontario, Canada	N= 155,448	Total physical activity measured in MET-hours/week, including vigorous, moderate, and	Physical activity was associated with lower OSA prevalence independent of BMI. For each increase in

apnea in a large, general population cohort study.[48]				walking activity; OSA assessed by self-report.	total physical activity (MET-hours/week), the odds of OSA decreased slightly: OR 0.98 (95% CI 0.96–1.00), p≤0.045. Similar associations for vigorous activity and walking: OR 0.98 (95% CI 0.96–1.00), p≤0.045.
Mônico-Neto, M., et al. (2018). "Physical activity as a moderator for obstructive sleep apnoea and cardiometabolic risk in the EPISONO study." [49]	Longitudinal cohort study	São Paulo, Brazil	N=658	Physical activity assessed using the International Physical Activity Questionnaire (IPAQ); active vs inactive.	Active participants had a lower risk of developing sleep apnea compared with inactive individuals (RR=0.877; 95% CI [0.296–0.855]). Among participants with apnea, being active halved the risk of developing type 2 diabetes vs inactive participants (RR=0.493; 95% CI [0.252–0.961]). Physical activity was associated with improved biomarkers: C-reactive protein (p=0.001), interleukin-6 (p=0.03), insulin (p=0.03), triglycerides (p<0.001), and insulin

					resistance (HOMA-IR) ($p<0.024$).

Table 1. Summary of clinical studies evaluating rehabilitation-based interventions in individuals with obstructive sleep apnea. The table describes study design, geographic location, sample characteristics, type of intervention, and main clinical outcomes, including respiratory indices, functional performance, sleep quality, and patient-reported outcomes. Interventions include aerobic and combined exercise training, inspiratory muscle training, oropharyngeal myofunctional therapy, and interdisciplinary lifestyle programs. Outcomes are reported using both polysomnographic measures and functional or symptom-based endpoints to reflect the multidimensional impact of rehabilitation in obstructive sleep apnea. Apnea–Hypopnea Index (AHI); Body Mass Index (BMI); Cerebral Metabolic Rate of Glucose (CMRgl); Control Group (CG); Effect Size (ES); Epworth Sleepiness Scale (ESS); Forced Expiratory Volume in One Second (FEV_1); Frequency, Intensity, Time, and Type (FITT); Functional Outcomes of Sleep Questionnaire (FOSQ); High-Density Lipoprotein (HDL); High-Intensity Interval Training (HIIT); Homeostatic Model Assessment of Insulin Resistance (HOMA-IR); International Physical Activity Questionnaire (IPAQ); Intervention Group (IG); Maximal Inspiratory Pressure (PImax); Maximum Heart Rate (HRmax); Mean Difference (MD); Metabolic Equivalent Task–hours per week (MET-hours/week); Muscle Sympathetic Nerve Activity (MSNA); Obstructive Sleep Apnea (OSA); Odds Ratio (OR); Peak Oxygen Uptake (VO_2peak); Peripheral Oxygen Saturation (SpO_2); Pittsburgh Sleep Quality Index (PSQI); Randomized Controlled Trial (RCT); Respiratory Event Index (REI); Sleep Apnea Quality of Life Index (SAQLI); Standard Deviation (SD); Standardized Mean Difference (SMD); Stroop Color and Word Test (SCWT); Training Heart Rate (HRtraining); Very Low-Density Lipoprotein (VLDL); Maximal Oxygen Uptake (VO_2max); 95% Confidence Interval (95% CI).
